# Comparison of patient characteristics and treatment approaches for femoral and inguinal hernias utilizing dynamic ultrasound at a single institution

**DOI:** 10.1007/s10029-023-02810-2

**Published:** 2023-05-30

**Authors:** M. K. Chiu, M. O. Hadied, C. Klochko, M. T. van Holsbeeck

**Affiliations:** 1 University of Southern California, 1500 San Pablo St., 2nd Floor Imaging, Los Angeles, CA 90033 USA; 2 Department of Radiology, Henry Ford Health System, 2799 W. Grand Boulevard, Detroit, MI 48202 USA

**Keywords:** Femoral hernia, Inguinal hernia, Ultrasound, Surgical repair

## Abstract

**Purpose:**

To assess the differences in management approach to femoral versus inguinal hernias and to identify patient characteristics associated with each hernia type.

**Methods:**

Imaging studies for patients who had undergone dynamic ultrasound evaluation for the symptom of groin pain between January 1, 2010, and March 31, 2019, at a single institution Musculoskeletal Department were analyzed. Positive femoral hernia imaging studies were compared to studies for inguinal hernias and matching medical records for imaging studies were analyzed. Association of patient characteristics (age, sex, smoking, diabetes) with hernia type was assessed. Primary outcomes were surgical versus non-surgical approach, type of surgery, number of follow-up visits, and pain resolution.

**Results:**

A total of 1319 patients presented with groin pain and were assessed with dynamic ultrasound (534 female; 785 male; mean [± SD] age 48.2 ± 16.5). While 409 (31.0%) patients had a femoral hernia detected, 666 (50.6%) had an inguinal hernia detected (*p* < .05). Significantly more inguinal hernias were surgically repaired than femoral hernias (65.0% vs 53.9% *p* = .008), and more inguinal hernias than femoral hernias were treated with open surgery (71.0% vs 57.7%; *p* = .014). Patients with femoral hernias had significantly more follow-up clinic visits than patients with inguinal hernias (mean [± SD] 2.65 ± 4.80 vs 1.76 ± 1.27; *p* = .010). No difference in the percentage of patients who had pain resolution was observed (82.2% inguinal vs 75.0% femoral; *p* = .13).

**Conclusions:**

Femoral hernias were managed more conservatively than inguinal hernias at our institution.

## Introduction

Although clinically apparent femoral hernias are less common than inguinal hernias, they are associated with higher rates of acute complications. One study found that cumulatively, femoral hernia strangulation occurred in 22% of patients 3 months after diagnosis and in 45% of patients after 21 months compared to only 3% and 4.5% for inguinal hernias, respectively [1]. Other studies have shown that acute femoral hernias and their subsequent complications are associated with increased morbidity and mortality, such as increased rates of bowel resection, wound infection, and cardiovascular and respiratory compromises [2–5]. One study found that emergent repair of femoral hernias was associated with a sevenfold increased risk of mortality and a 20-fold increase if concomitant bowel resection was performed [6].

Hernias can be diagnosed utilizing various radiology modalities including ultrasound, computed tomography and magnetic resonance imaging. Evaluation of femoral hernias diagnosed by the less invasive and more sensitive ultrasound approach are scant. Because the literature suggests that femoral hernias may carry a higher risk of unfavorable outcomes, and because dynamic ultrasound is a very sensitive approach for diagnosing inguinofemoral hernia (reported 95% sensitivity in the literature), we hypothesized that a surgical approach might be more common for patients with femoral hernia identified by ultrasound compared to inguinal hernia [7]. Therefore, we assessed whether treatment approach, patients’ characteristics, and outcomes were associated with the type of ultrasound-detected hernia. In particular, our aim was to shed light on treatment trends for patients with the less common femoral hernia within the context of the more accurate diagnostic approach of dynamic ultrasound. Understanding trends of treatment approach and patient outcomes may help refine best clinical approaches for this uncommon condition.

## Methods

### Patients

**Fig. 1:**
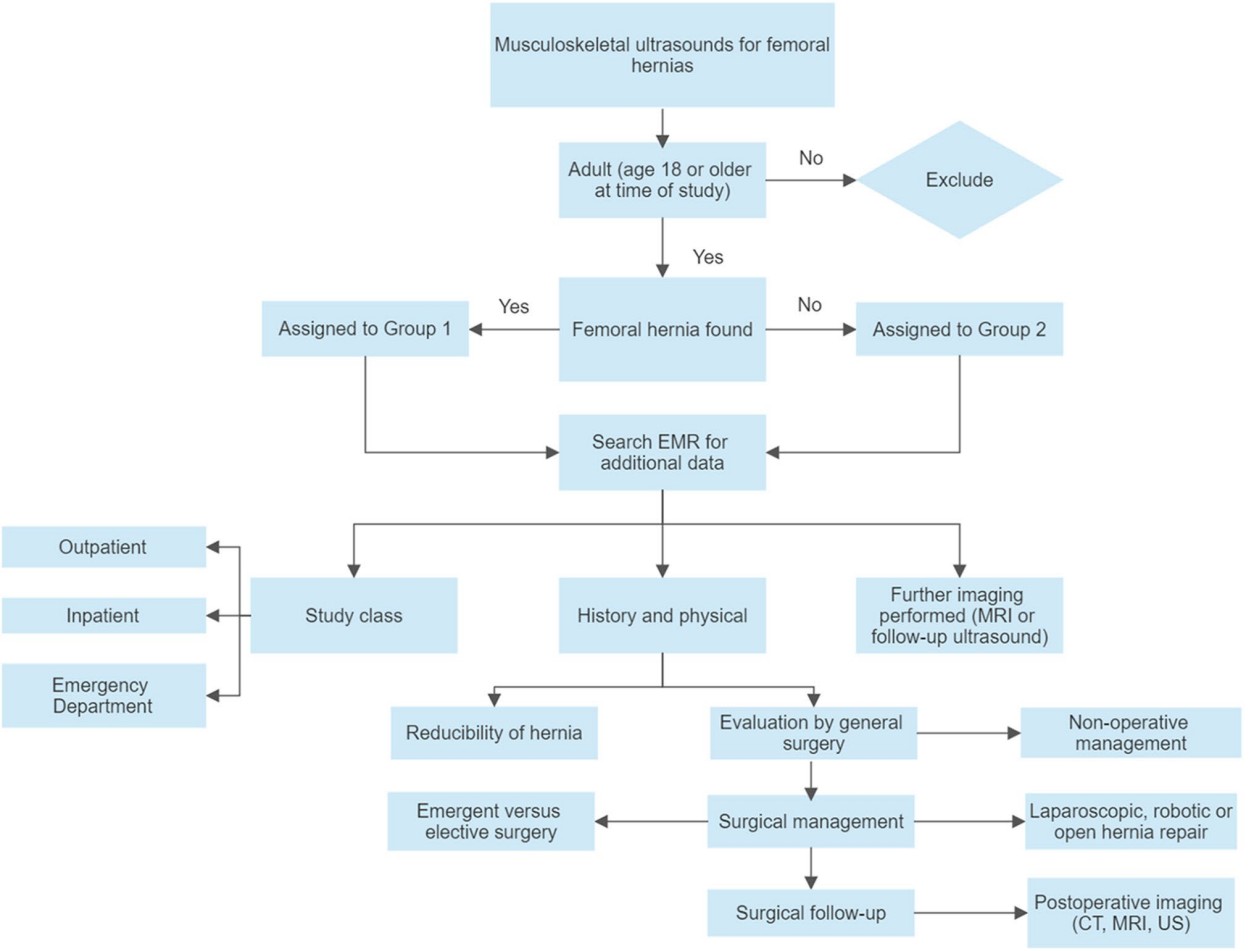
Flowchart demonstrating variables of interest during data acquisition

This single institution retrospective study included patients who were evaluated by dynamic ultrasound in the Musculoskeletal Department between January 1, 2010, and March 31, 2019. The study obtained approval from the Institutional Review Board of the sponsoring institution. The need for informed consent was waived. The authors ran a preliminary query for the phrase “femoral hernia” within the picture archive and communication system (PACS) dictations for ultrasounds that were performed by the Musculoskeletal Department for the indication of groin pain; this returned 1450 study-specific accession numbers for adult patients (age 18 years or older at the time of study). As part of department protocol, evaluation for femoral hernias also included evaluation for inguinal hernias so a separate query was not performed. From this population, one accession number was excluded because of incomplete data, and the remaining 1449 accession numbers resulted in 1319 unique patients (Fig. 1).

### Image acquisition and data processing

**Fig. 2:**
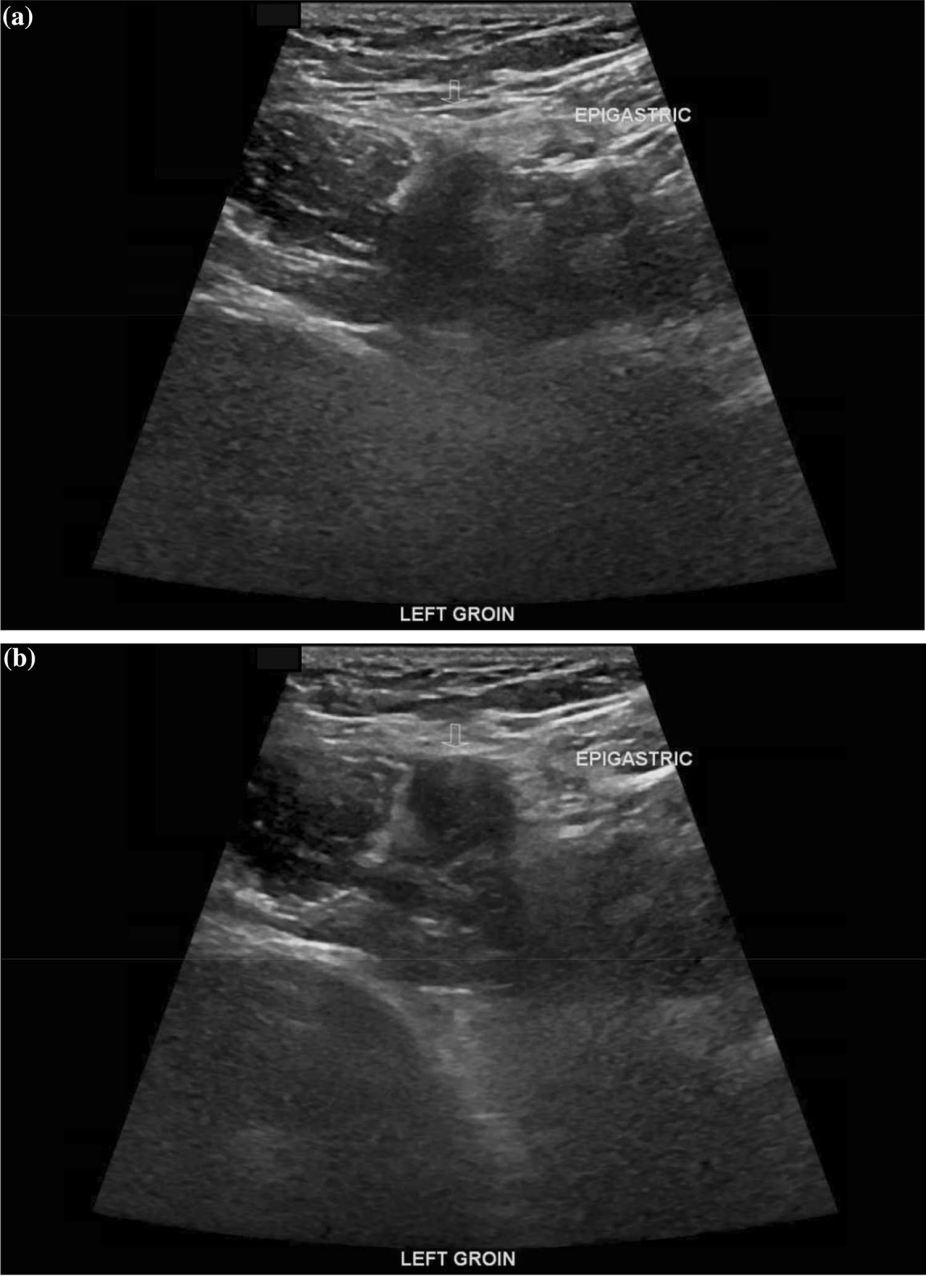
Ultrasound images demonstrating soft tissue protrusion medial to the epigastric vessels, labeled as epigastric in the image, within the inguinal canal in the left groin acquired pre-(**a**) and post (**b**)-valsalva. Open arrow indicates region of interest for soft tissue protrusion

**Fig. 3:**
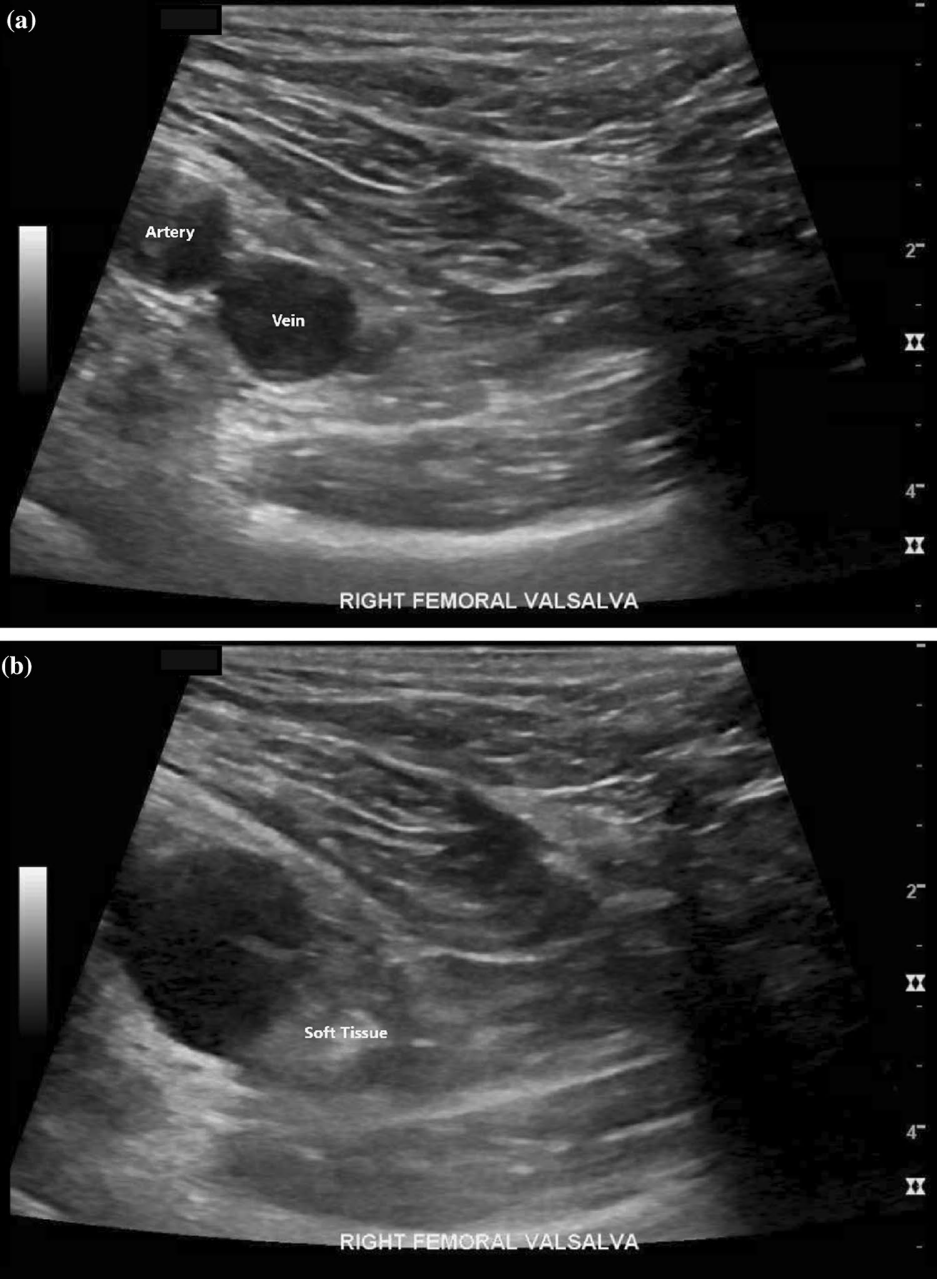
Ultrasound images demonstrating hyperechoic soft tissue protrusion medial to the femoral artery and vein within the femoral canal acquired pre-(**a**) and post (**b**)-valsalva. Femoral artery in the image is labeled as artery, and the femoral vein is labeled as vein

Images had been obtained following the institutional musculoskeletal protocol to evaluate for groin pain, which included evaluating the hip joint, and assessing for inguinal (Figs. 2) and femoral hernias (Figs. 3) with GE Logiq E9 ultrasound machines [8, 9]. Patients were scanned by sonographers certified by the American Institute of Ultrasound in Medicine to perform musculoskeletal ultrasound examinations, reviewed by a musculoskeletal radiologist and rescanned if necessary. Final interpretations of imaging studies were dictated by 8 musculoskeletal-trained radiologists over the study period, each with at least a decade experience in interpreting musculoskeletal ultrasounds with experience ranging from 10 to 30 years.

Institutional protocol to evaluate groin pain proceeded as follows: the transducer was placed transversely over the patient in supine position to identify the origin of the inferior epigastric artery. A Valsalva maneuver was taught to the patient, and adequacy determined by the degree of visual distension of the femoral and iliac veins. Criteria to diagnose hernia was presence of movement of soft tissue—whether it be bowel, bladder, lipomatous tissue in continuation with the abdominal cavity, or any combination of the three, with or without Valsalva maneuver. Presence of tissue moving medially or laterally with repetitive Valsalva maneuvers relative to the inferior epigastric vessels was utilized to diagnose direct or indirect inguinal hernias. The transducer was then moved over to the superior pubic ramus to identify the pectineus muscle adjacent to the femoral vein. Valsalva maneuver was repeated to identify presence or absence of soft tissue protrusion on dynamic ultrasound to diagnose a femoral hernia. Then, the transducer was repositioned transversely over the obturator canal and external obturator muscle to examine for obturator hernias. Additionally, each study evaluated the external hip capsule and pubic symphysis to exclude non-hernia causes of groin pain. If abnormality was found in the hip capsule or pubic symphysis, Marcaine test injection was performed to see if that resolved the patient’s pain. Further details are described in a previous publication [10].

The electronic medical record (EPIC) was accessed to evaluate for surgical evaluation and any subsequent treatment of the patient population. Patient location (outpatient, inpatient, or Emergency Department) and evaluation by general surgery with subsequent decision for non-surgical (conservative) versus surgical management were assessed. For patients who underwent operative care, the type of surgery (open, laparoscopic, and robotic) was compared, and follow-ups utilizing the electronic medical record during the study timeframe.

### Statistical analysis

Nominal data were compared using the Chi-square statistic while continuous data were examined using two-sample Wilcoxon statistics to evaluate the management of femoral and inguinal hernias. A nonparametric Wilcoxon test was used because the data were not normally distributed. A two-tailed *p* value ≤ 0.05 was considered statistically significant. All statistical tests were performed using SAS Version 9.1.

## Results

A total of 1319 patients (534 female, 785 male; mean [± SD] age 48.2 ± 16.5 years) were included in the analysis. There were 409 femoral hernias and 666 inguinal hernias that were identified by dynamic ultrasound within the Musculoskeletal Radiology Department between January 1, 2010, and March 31, 2019 for a given indication of groin pain. The female-to-male ratio was 1.08:1 for positive femoral hernias and 0.41:1 for inguinal hernias. Approximately 99.2% of all examinations were performed in the outpatient setting, while the others were done in the emergency (0.7%) and inpatient (0.2%) settings. Patient baseline characteristics are shown in Table 1. Of note, patients with inguinal hernias had a higher rate of diabetes than those with femoral hernias (11.6% vs 6.5%; *p* = 0.015). (Table 2).

**Table 1:** Characteristics of patients who presented with femoral or inguinal hernia stratified by patient sex

Variable	All patients N = 1319	Female patients (n = 534)	Male patients (n = 785)	*p* value^a^
Age, years	48.2 ± 16.5	48.0 ± 15.7	48.3 ± 17.1	0.72
Femoral hernia				
Total	31.0 (409/1319)	39.7 (212/534)	25.1 (197/785)	0.001
Right side	61.0 (244/400)	62.1 (128/206)	59.8 (116/194)	0.63
Left side	23.0 (92/400)	23.3 (48/206)	22.7 (49/194)	0.88
Bilateral	16.0 (64/400)	14.6 (30/206)	17.5 (34/194)	0.43
Inguinal hernia	50.5 (666/1317)	36.3 (193/532)	60.3 (473/785)	0.001
Femoral and inguinal hernia	17.4 (229/1317)	17.9 (95/532)	17.1 (134/785)	0.71
Study location				
Outpatient	99.2 (1308/1319)	99.3 (530/534)	99.1 (778/785)	0.78
Inpatient	0.2 (2/319)	0.2 (1/534)	0.1 (1/785)	1.00
Emergency	0.7 (9/1319)	0.6 (3/534)	0.8 (6/785)	0.74
Smoker	38.2 (446/1167)	35.2 (163/463)	40.2 (283/704)	0.09
Diabetes	9.2 (107/1170)	8.6 (40/463)	9.5 (67/707)	0.63

^a^Data are reported as mean ± standard deviation or percentage (r/n). A comparison of age with the two sample Wilcoxon test, all others used Chi-squared test

**Table 2:** Comparison of patient characteristics to presence of femoral and inguinal hernia

Variable	Femoral hernia N = 409	Inguinal hernia N = 437	*p* value^a^
Smoker	41.8%	38.9%	0.409
Diabetes	6.5%	11.6%	0.015
Patient age, years, mean ± SD			
All patients	49.7 ± 15.4	49.0 ± 17.0	0.607
Female	50.3 ± 15.0	47.7 ± 16.0	0.189
Male	49.0 ± 15.7	49.3 ± 17.3	0.765

^a^Two sample Wilcoxon tests were used to compare groups

### General surgery evaluation and management

From the patient population, 709/1319 (53.8%) patients were referred to general surgery for hernia evaluation and management. A total of 140/260 patients (53.9%) with femoral hernias underwent surgical repair, while 186/286 (65.0%) patients with inguinal hernias underwent surgical repair (*p* = 0.008). No significant difference in femoral versus inguinal hernia repair rates was observed for women (*p* = 0.88); however, in men, 68/131 (51.9%) femoral hernias were surgically repaired compared to 149/221 (67.4%) inguinal hernias (*p* = 0.004). Additionally, significantly more inguinal hernias than femoral hernias were repaired by open surgery (57.7% femoral vs 71% inguinal; *p* = 0.014) than laparoscopic (23.9% femoral vs 24.6% inguinal; *p* = 0.11) and robotic approaches (9.5% femoral vs 4.5% inguinal; *p* = 0.08). (Table 3). The majority of the ultrasound results correlated with the operative findings, with only 21/140 (15%) of surgeries with positive ultrasound resulting in negative operative finding. There were 4/186 (2.2%) of inguinal surgeries that found femoral hernias which were described as negative on ultrasound.

**Table 3:** Comparison of clinical care given to patients with femoral versus inguinal hernia

Variable	Femoral hernia (n = 401)	Inguinal hernia (n = 435)	*p* value^a^
Surgical repair			
Open	57.7%	71.0%	0.014
Laparoscopic	23.9%	24.6%	0.11
Robotic	9.5%	4.5%	0.08
Non-surgical care	6.5%	11.6%	0.015
Postoperative imaging, no. (%)			
All imaging	8.7% (35/401)	6.0% (26/435)	0.13
Magnetic resonance imaging	34.4%	42.3%	0.54
Computed tomography	25.0%	50.0%	0.049
Ultrasound	50.0%	26.9%	0.07
Surgical hernia repair			
All surgeries	53.9%	65%	0.008
Female	55.8%	56.9%	0.88
Male	51.9%	67.4%	0.009

^a^Chi-squared test was used to compare all groups

### Postoperative care and outcomes

In the postoperative setting, patients who had femoral hernia repair had a significantly higher number of follow-up visits than patients who received inguinal hernia repair (mean [± SD] 2.65 ± 4.80 vs 1.76 ± 1.27; *p* = 0.010). However, no significant difference was seen in resolution of postoperative pain for either type of hernia (75.0% femoral vs 82.2% inguinal; *p* = 0.13) (Table 4). Further postoperative imaging to evaluate for recurrence was done with a combination of ultrasound, computed tomography (CT), and magnetic resonance imaging (MRI). A significant preference for using CT to evaluate inguinal hernias was observed (50.0% inguinal vs 25.0% femoral; *p* = 0.049), while no significant difference was evident in the use of MRI or ultrasound for evaluating inguinal versus femoral hernias (Table 3).

**Table 4:** Comparison of patient postoperative outcomes after femoral inguinal hernia repair

Outcome	Femoral hernia (n = 128)	Inguinal hernia (n = 174)	*p* value^a^
Number of follow-up visits, mean ± SD	2.65 ± 4.80	1.76 ± 1.27	0.010
Pain resolution (%)	75.0%	82.2%	0.130

^a^Two sample Wilcoxon tests were used to compare groups

## Discussion

In this study, we showed that ultrasound-detected femoral hernias were managed more conservatively than inguinal hernias at our institution, where patients with inguinal hernias were more likely to have received surgery. In particular, men were more likely to have surgery performed for inguinal hernia, while women were equally likely to have surgery for either type of hernia. Additionally, in contradistinction to previously reported literature, the female-to-male ratio for femoral hernias that we observed was low, at 1.08:1, compared to 3:1 (NHDS), 3.13:1 (ACS-NSQIP), and 1.67:1 (Swedish Hernia Register**)** [11–13].

Despite the high rates of associated morbidity and mortality from femoral hernias reported in the surgical literature, data from our institution showed that ultrasound-detected femoral hernias were treated more conservatively than inguinal hernias. We observed that a significantly smaller percentage of patients were evaluated by surgeons and subsequently went on to operative management for femoral hernia than patients with inguinal hernia, especially men. Additionally, we found that in the postoperative setting, patients who had undergone femoral hernia repair had a higher number of follow-up clinic visits than those who had undergone inguinal hernia repair, which may suggest increased complexity in patients with femoral hernias.

Our research provides some contrast to the previously reported literature on femoral hernias. Beadles and colleagues found that emergent hernia repair rates for femoral hernias were higher than for inguinal hernias [13], whereas our study found that the treatment and management approach for femoral hernias at our institution was more conservative than the approach for inguinal hernias. There may be several reasons for this apparent contradiction, with one important distinction between our study and previous studies being the difference in patient populations. The majority of our patients were classified as outpatients for evaluation of groin pain thought to be musculoskeletal pain in etiology as opposed to inpatients or emergency admissions who may present with gastrointestinal symptoms. It is feasible that the patient population we captured may vary drastically from the patients who present to the Emergency Department for evaluation of abdominal or obstructive symptoms. This may also account for the difference we saw in the prevalence of femoral hernias detected in women versus men compared to previously published literature.

Because ultrasound is an extremely sensitive diagnostic modality, with high positive predictive value for detection of inguinal and femoral hernias, it is possible that the femoral hernias detected in our study may have been incipient hernias or otherwise clinically silent conditions that might have remained undiscovered in the absence of ultrasound analysis [7, 14–16]. It is worth noting that in the electronic medical record, some surgeons had specifically commented that small hernias were felt to be clinically silent. Additionally, Alabrabra et al. found a positive predictive value of 70% when comparing ultrasound-detected groin hernias to those found by surgical exploration, indicating that in 30% of cases, a positive ultrasound finding may have resulted in a negative groin exploration operation [15]. This is in line with findings reported by Brandel et al. who reported a positive predictive value of 71% and negative predictive value of 92% [16]. Thus, ultrasound-detected femoral hernias may indicate a clinically insignificant finding rather than a pressing need for surgical correction [12].

As alluded to previously, our study has several limitations. The first is that the patient population may have differed from those evaluated in the surgical literature in regard to signs and symptoms for femoral hernias. Because our population was predominantly composed of outpatient studies, there may have been an inherent selection bias of selecting healthier patients rather than sicker patients who present to the Emergency Department. Another factor to consider is that our study evaluated patients who presented with groin pain, which may have a multitude of causes, including labral or muscle tears, whereas patients who present to the Emergency Department with incarcerated and/or strangulated hernias may have obstructive symptoms such as vomiting or inability to pass flatus.

In summary, our study found that ultrasound-detected femoral hernias were managed more conservatively than inguinal hernias at our institution, and that the prevalence of femoral hernias in women compared to men was lower than the rates that have been reported in the surgical literature. Reasons for the difference in management approach and prevalence may be attributed to our unique patient population, the possible detection of incipient or clinically silent femoral hernias, and other factors. Despite the limitations of the study, our research does raise an interesting question as to whether, given long enough follow-up, ultrasound-detected femoral hernias might eventually become clinically apparent. A prospective longitudinal study of patients with groin pain and asymptomatic femoral hernias could shed further light on the significance of our findings.

## Data Availability

Not applicable.
